# A Novel Heterogalactan from *Antrodia camphorata* and Anti-Angiogenic Activity of Its Sulfated Derivative

**DOI:** 10.3390/polym9060228

**Published:** 2017-06-16

**Authors:** Yanqiu Liu, Yaqi Ding, Min Ye, Tao Zhu, Danbi Tian, Kan Ding

**Affiliations:** 1 Glycochemistry and Glycobiology Lab, Shanghai Institute of Materia Medica, Chinese Academy of Sciences, 555 Zu Chong Zhi Road, Shanghai 201203, China, University of Chinese Academy of Sciences, No. 19A Yuquan Road, Beijing 100049, China; lyanqiu@mail.ustc.edu.cn (Y.L.); 3481918100@njtech.edu.cn (Y.D.); 2 Nano Science and Technology Institute, University of Science and Technology of China, 96 Jin Zhai Road, Hefei 230026, China; 3 Department of Chemical and Molecular Engineering, Nanjing Tech University, 30 Puzhu South Road, Nanjing 211816, China; 4 State Key Laboratory of Natural and Biomimetic Drugs, School of Pharmaceutical Sciences, Peking University, 38 Xueyuan Road, Beijing 100191, China; yemin@bjmu.edu.cn

**Keywords:** *Antrodia camphorata*, mannofucogalactan, sulfated polysaccharide, anti-angiogenesis

## Abstract

A heterogalactan, named ACW0, was extracted from *Antrodia camphorata* and purified by anion exchange and gel permeation chromatography. It was composed of galactose (94.98%), traces of mannose (2.41%), and fucose (2.61%), with its molecular weight estimated to be 13.5 k Da. The polysaccharide ACW0 was shown to be a mannofucogalactan with a backbone chain of α-d-1,6-linked Gal, attached by a non-reducing terminal α-d-Man and α-l-Fuc on C-2 of nearly every six α-d-1,6-linked Gal residues. A sulfated polysaccharide, ACW0-Sul was achieved by the chlorosulfonic acid-pyridine method. Compared with the native polysaccharide, ACW0-Sul could disrupt tube formation and migration as well as cell growth of human microvascular endothelial cells (HMEC-1) dose-dependently. Further studies revealed that phosphorylation of Extracellular Regulated Protein Kinases (Erk) and Focal Adhesion Kinase (FAK) were significantly inhibited by ACW0-Sul. These results suggested that ACW0-Sul could be a potent candidate for anti-angiogenic agent development.

## 1. Introduction

Angiogenesis, the development of new blood vessels from existing vessels, occurs mainly during human development and reproduction. This process of angiogenesis promoting factors and angiogenesis inhibitory factors [1] and the imbalance of the two types of regulation lead to abnormal blood vessels, causing disease. Embryonic development, wound healing, and female physiological cycles are inseparable from the critical aspects of angiogenesis under normal circumstances, and they are subject to strict control and regulation in the body. When the body has problems, resulting in loss of regulation and control of angiogenesis, it leads to related diseases, such as the occurrence and development of a variety of tumors and metastases [2], and diabetes [3].

Tumor angiogenesis refers to the process of growing new blood vessels from existing vascular beds, involving a series of complex specificities such as increased cell numbers and cell migration. Tumor cells can grow indefinitely, grow self-reliably, can avoid the death signal, and have a strong ability to organize invasion and metastasis, which requires the existence of sustained angiogenesis in tumor tissue, the need for endothelial cells, tumor cells, marginal cells and vascular smooth muscle cells. Therefore, controlling tumor-associated angiogenesis has become a promising tactic in limiting cancer progression [4]. Our group has reported that sulfated polysaccharide WSS25 [5], FP08S2 [6], and NDH01 [7] inhibited angiogenesis by binding to different proteins. It is essential to search for safer and effective potential drugs to ensure this development.

*Antrodia camphorate*, a rare and expensive mushroom, native to Taiwan, has been considered as having diverse medicinal benefits for the treatment of poisoning, drug intoxication, diarrhea, abdominal pain, hypertension, skin itches, and liver cancer [8]. It has been reported that various bioactivities may be associated with this mushroom-derived polysaccharide. In recent years, numerous bioactivity effects of the polysaccharide from *A. camphorata* have aroused the attention of researchers, including antioxidant [9], hepatoprotective [10], antiangiogenic [11], anticancer [12] and anti-inflammatory properties [13].

However, the different biological activities of the polysaccharides rely on features of their structure, including monosaccharide composition, linkage type, configuration and even comformation. Most studies have revealed that classic heterogalactan from mushroom has a backbone of 1,6-linked α-d-galactopyranosyl residues, which are substituted at the C-2 atom position either with l-fucpyranose or 3-*O*-α-d-mannopyranosyl-α-l-fucopyranosyl residues [14,15,16,17,18]. The Chia-Chuan Chang group reported that a fucosylated 1,6-α-d-mannogalactan from *A. camphorata* exhibited anti-angiogenic bioactivity [15]. Additionally, there is some evidence that chemical modification of polysaccharides can generate stronger or new bioactivities in comparison with native polysaccharide [19,20,21,22]. In our laboratory, we found that heteroxylan from *Cassia obtusifolia* after sulfation [23] showed significant inhibition on tube formation of human microvascular endothelial cells and on the growth of Bel7402 liver cancer cells. However, no bioactivity was observed when using native polysaccharide treated cells (HMECs). The biological activities of sulfated polysaccharides correlated with the degree of sulfation, the sulfation position, the molecular weight etc. Thereby, the chemical structures of sulfated polysaccharides with relevance to their biological activities is worthy of study. To further investigate the functional mechanism underlying the action of sulfated polysaccharide, the relationship between chemical structures and bioactivities of the different polysaccharides should be clearly elucidated. In this study, the structure of polysaccharide from *A. camphorata* was characterized and the anti-angiogenic bioactivity of its sulfated polysaccharide was also evaluated.

## 2. Materials and Methods

### 2.1. Materials

The lyophilized powders of *A. camphorata* mycelium were obtained from Dr. Min Ye in the School of Pharmaceutical Sciences, Peking University, Beijing, China. Diethylaminoethyl (DEAE) sepharose Fast Flow and Sephacryl S-100HR were commercially purchased from GE Healthcare Life Science. T-series Dextrans were obtained from Amersham Pharmacia Biotech (Little Chalfont, Buckinghamshire, UK). Monosaccharide standards were all purchased from Fluka, Switzerland. Dimethyl sulfoxide (DMSO) was obtained from Merck (Darmstadt, Germany), whereas 3-(4,5-dimethylthiazol-2-yl)-2,5-diphenyltetrazoliumbromide (MTT) came from Sigma-Aldrich, St. Louis, MO, USA. Sodium borohydride (NaBH_4_), iodomethane (CH_3_I), and trifluoroacetic acid (TFA) were all from Sinopharm Chemical Reagent Co. Ltd. Other reagents were analytical grade unless otherwise stated.

### 2.2. General Methods

IR spectra were conducted by employing KBr pellets for natural polysaccharide or Nujol film for permethylated polysaccharide. Optical rotation was determined with an Autopol VI instrument in distilled water at 20 °C and wavelength of 589 nm. High performance gel permeation chromatography (HPGPC) on an Agilent 1260 HPLC system equipped with series-connected Ultrahydrogel 2000 and 500 columns was used for homogeneity and molecular weight evaluation. GC-MS was performed on a Shimadzu QP2010 Plus apparatus. The sulfate content was measured by the barium chloride gelatin method [24].

### 2.3. Isolation and Purification of Polysaccharide from A. Camphorata

The lyophilized powder of *A. camphorata* mycelium was extracted with boiling water 4 times (4 h for each extraction). After centrifugation, the supernatant was condensed and dialyzed against running water for 3 days. Then the retentate was concentrated and precipitated in 70% EtOH, overnight. The precipitate was washed with absolute ethanol and acetone, followed by drying under vacuum at 45 °C to yield the crude polysaccharide. Furthermore, DEAE sepharose Fast Flow and Sephacryl S-100HR systems were employed to obtain the target polysaccharide ACW0.

### 2.4. Purity and Molecular Weight Determination

Homogeneity and molecular weight were determined on an Agilent 1260 HPLC system equipped with series-connected Ultrahydrogel 2000 and 500 columns [25] with UV and RI as detectors at a flow rate of 0.5 mL/min, eluted with 0.1 M NaNO_3_ as mobile phase. The sample was prepared as a 0.4% (*w*/*v*) solution followed by analysis with 20 μL of solution injected in each run. The column was kept at 25 °C.

### 2.5. Monosaccharide Composition Analysis

ACW0 monosaccharide composition was analyzed by GC as alditol acetate according to a previous method [26]. In brief, ACW0 was hydrolyzed with 2 M TFA, reduced with NaBH_4_, and acetylated with acetic anhydride to obtain the final alditol acetate derivate before analysis.

### 2.6. Glycosyl Linkage Analysis

For complete methylation, the polysaccharide was first methylated twice with Haworth’s method [27] and afterwards methylated three times with the modified Ciucanu’s method [28]. The methylated polysaccharide was further transformed into the partially methylated alditol acetate and analyzed by GC-MS.

### 2.7. NMR Analysis

An amount of 40 mg of polysaccharide was deuterium-exchanged overnight and dissolved again in 0.5 mL in D_2_O. ^1^H NMR, ^13^C NMR, ^1^H-^1^H correlation spectroscopy (^1^H-^1^H COSY), hetero-nuclear single quantum coherence (HSQC), and hetero-nuclear multiply bond correlation (HMBC) spectra were measured on a Bruker AVANCE III NMR spectrometer operating at 500 MHz with acetone as internal standard (31.5 ppm for carbon, 2.26 ppm for hydrogen).

### 2.8. Preparation of Sulfated Derivatives

The sulfation of polysaccharide was performed using the chlorosulfonic acid-pyridine method [29]. Briefly, sulfating reagent was first made from chlorosulfonic acid-pyridine (*v*/*v*, 2:1) and then added to polysaccharide (50 mg), which was dissolved in 2.5 mL of dried formamide in an ice bath. The mixture was maintained at 40 °C for 4 h under continuous stirring and then neutralized with 5 M NaOH. The solution was first dialyzed with saturated NaHCO_3_, then against distilled water. Finally, the retentate was lyophilized to obtainthe final sulfated derivate ACW0-Sul. The degree of sulfation (DS), which is the average number of sulfate groups on each sugar residue, was calculated from the sulfur content based on the barium chloride gelatin method [30], using the following formula:

DS = 162 × S% (32 − 102 × S%)

### 2.9. Cell Lines and Culture Conditions

Human microvascular endothelial cells (HMEC-1) were maintained in MCDB131 (Gibco BRL, Pittsburgh, PA, USA) medium containing 15% FBS (Sijiqing Co., Ltd., Hangzhou, China), 2 mM l-glutamine, and 10 ng/mL Epidermal Growth Factor (EGF) (Shanghai Prime Gene Bio-Tech Co. Ltd., Shanghai, China). The cells were cultured in an incubator at 37 °C under a hygric atmosphere containing 5% CO_2_ as mentioned in previous reports [4].

### 2.10. Tube Formation on Matrigel

The anti-angiogenesis activities of ACW0 and ACW0-Sul were detected by capillary-like tube formation assay in vitro. The process was conducted as described in previous reports [4]. The anti-angiogenesis effects of ACW0 (0, 9.25, 18.5, 37, and 74 μM) and ACW0-Sul (0, 3.5, 7, 14, and 28 μM) were examined respectively and all other conditions. The HMEC-1 (5 × 10^5^ cells per well) cells seeded on top of the matrigel-coated (50 μL per well) wells of 96-well plates were composed of ACW0 (0, 9.25, 18.5, 37, and 74 μM) and ACW0-Sul (0, 3.5, 7, 14, and 28 μM); without ACW0 or ACW0-Sul only the same amount of medium and cells as the control. The plate was then incubated at 37 °C and the formation of the capillary-like tubes was observed for another 12 h. The wells were photographed at 40× magnification by a microscope (IX 51, Olympus Imaging). Quantification of capillary-like tube formation in each well was analyzed by Image-J software (National Institutes of Health, Stapleton, NY, USA).

### 2.11. Wound Healing Assay of HMEC-1 Cells

HMEC-1 cell migration was detected by using a wound healing assay. As in previous reports [4,7], in short, 5 × 10^5^ cells/well were seeded into a 6-well plate. After 24 h incubation, the syncretic monolayer was nicked by an artificial cross-shaped wound in each well with a suitable force. Then after the cells were rinsed lightly with PBS three times, a new medium containing a different concentration of ACW0-Sul was provided. Meanwhile, the medium in the absence of ACW0-Sul served as control, followed by photographing. Then the cells were incubated for another 12 h. The wound area was examined by microscope and new photos were taken. Finally, comparing to the control, the inhibition rates were calculated quantitatively by Image J software.

### 2.12. Cell Proliferation (MTT) Assay

In brief, HMEC-1 cells (4 × 10^3^ cells/well) were seeded into a 96-well plate and incubated overnight. Then the cells were incubated with or without different ACW0-Sul (0, 0.4375, 0.875, 1.75, 3.5, 7, 14, and 28 μM) for 24, 48, and 72 h, respectively. After different times (24, 48, and 72 h), MTT (10 μL/well) was added and the cells were cultured at 37 °C for another 4 h. Then DMSO (150 μL/well) was added to the cells to sufficiently dissolve the purple formazan product. The absorbance was recorded at 490 nm using a Bio-Rad 3350 micro plate reader. Finally, the effect of ACW0-Sul on HMEC-1 cell viability was counted based on the percentage of control, which was randomly distributed at a value of 100% cell viability.

### 2.13. Western Blotting

Total protein samples were extracted by lysing cells with an equivalent Radio Immunoprecipitation Assay (RIPA) buffer (Beyotime, Haimen, China). The total cellular protein samples were separated by electrophoresis on SDS-PAGE gels and transferred to a polyvinylidene difluoride (PVDF) membrane (Biorad). The membrane was blocked with 5% nonfat milk or BSA in TBST buffer at room temperature for 2 h, and incubated with primary antibodies raised against phosphor-Erk, total-Erk, phosphor-FAK, total-FAK, and GAPDH (Cell Signaling Technology, Inc., Boston, MA, USA) at 4 °C, with sufficient contact overnight. Then the membrane was incubated with secondary antibodies. The blots were measured and visualized by enhanced chemiluminescence (ECL) reagent, followed by ImageJ software quantification and normalization.

### 2.14. Statistical Analysis

All results are presented as mean values ± standard error. Values of *p* < 0.05 showed statistically significant (* *p* < 0.05, ** *p* < 0.01, *** *p* < 0.001) [4]. Statistical analyses were carried out by *t*-test for comparison of two groups or variance analysis for multiple comparisons using GraphPad software (GraphPad Software, Inc., La Jolla, CA, USA).

## 3. Results

### 3.1. Isolation and Purification of Polysaccharide

An amount of 66.5 g crude polysaccharide (AC) (yield 6.65%) was obtained from 1 kg lyophilized powder of *A. camphorata* mycelium. The crude polysaccharide was subjected to DEAE sepharose Fast Flow column to give H_2_O eluate ACW (yield 0.62%). ACW was further purified by a Sphacryl S-100HR column to achieve the final fragment, designated as ACW0 (yield 0.18%). The total sugar amount in ACW0 was estimated to be 64.47% based on the phenol-sulfuric acid method. In addition, trace amounts of uronic acid and protein were included, respectively.

### 3.2. Composition and Linkage Analysis

A single symmetrical peak appeared on HPGPC indicating the high homogeneity of ACW0. Meanwhile, the molecular weight of this polysaccharide was estimated to be 13.5 k Da. Based on monosaccharide composition, ACW0 predominantly contained galactose (94.98%), traces of mannose (2.41%), and fucose (2.61%). The above results showed that ACW0 was likely to be a mannofucogalactan. The heterogalactan was methylated by Haworth’s method and modified Ciucanu’s method [31]. After hydrolysis and derivatization, the partially methylated alditol acetates were analyzed by GC-MS. The identification of all peaks was made by mass fragments in EI-MS and the characteristic retention times in GC. Integration of all corresponding peaks generated the molar ratios of the methylated sugars. The results are summarized in Table 1. Two major methylated sugars were the residues 1,6- and 1,2,6-linked Galp, in an approximate molar ratio of 3:1. Low amounts of fucose (8.9%) and mannose (5.38%) appeared in the non-reducing terminal forms. Together with monosaccharide composition, the results suggested that the polysaccharide has a backbone chain of 1,6-linked galactose with branches substituted at O-2 of the 1,2,6-linked galactose residues.

### 3.3. IR and Specific Rotation Analysis

**Figure 1:**
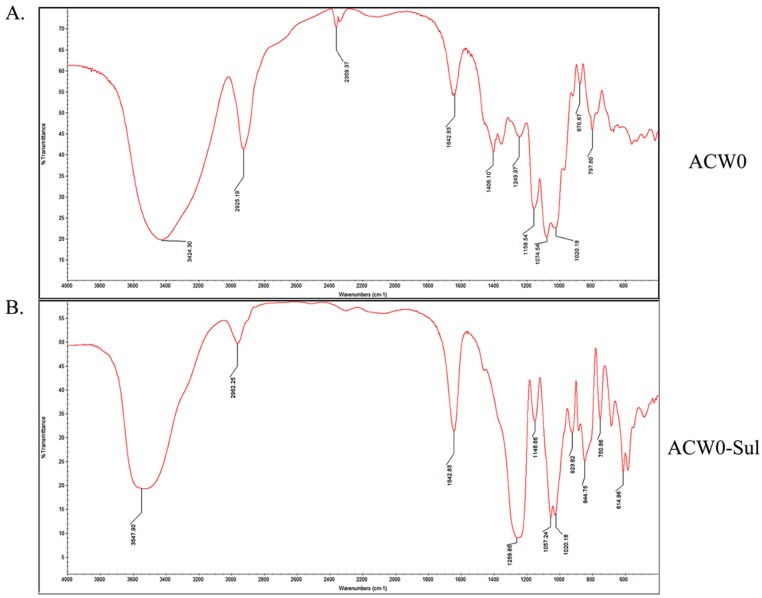
IR spectra of ACW0 (**A**) and ACW0-Sul (**B**).

The IR spectrum is shown in Figure 1. Comparing with native polysaccharide ACW0 (Figure 1A) there are two strong absorption bands at 1259.85 cm^−1^ and 844.75 cm^−1^ attributed to S=O and C–O–S stretching vibration of the sulfated polysaccharide ACW0-Sul (Figure 1B), which suggested the native ACW0 was successfully sulfated. The specific rotation of ACW0 was ${\left[\alpha \right]}_{D}^{25}$ and 85.5° (*C* = 0.1 g/100 mL).

### 3.4. 1D and 2D NMR Analysis

**Figure 2:**
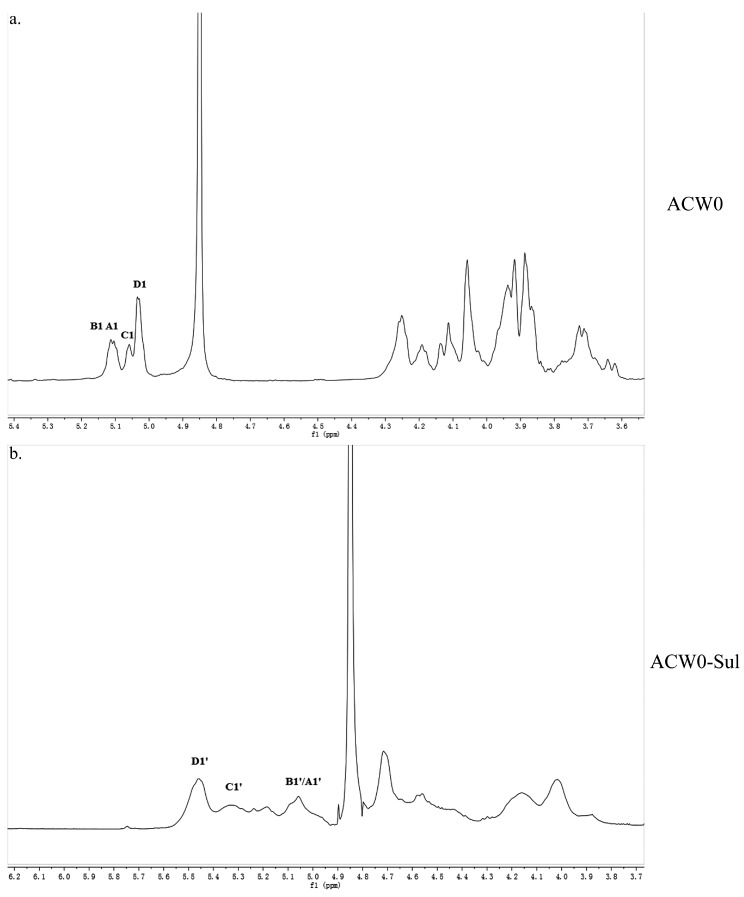
^1^H NMR spectrum of ACW0 (**a**) and ACW0-Sul (**b**) “A1” means terminally linked α-d-Man; “B1” means terminally linked α-l-Fuc; “C1” means 1,2,6-linked α-d-Gal; “D1” means 1,6-linked α-d-Gal. B1’/A1’, terminal Man sulfated at O-6; C1’, 1,2,6-linked Gal sulfated at O-3 and/or O-4; D1’, 1,6-linked Gal sulfated at O-2, O-3, and/or O-4.

**Figure 3:**
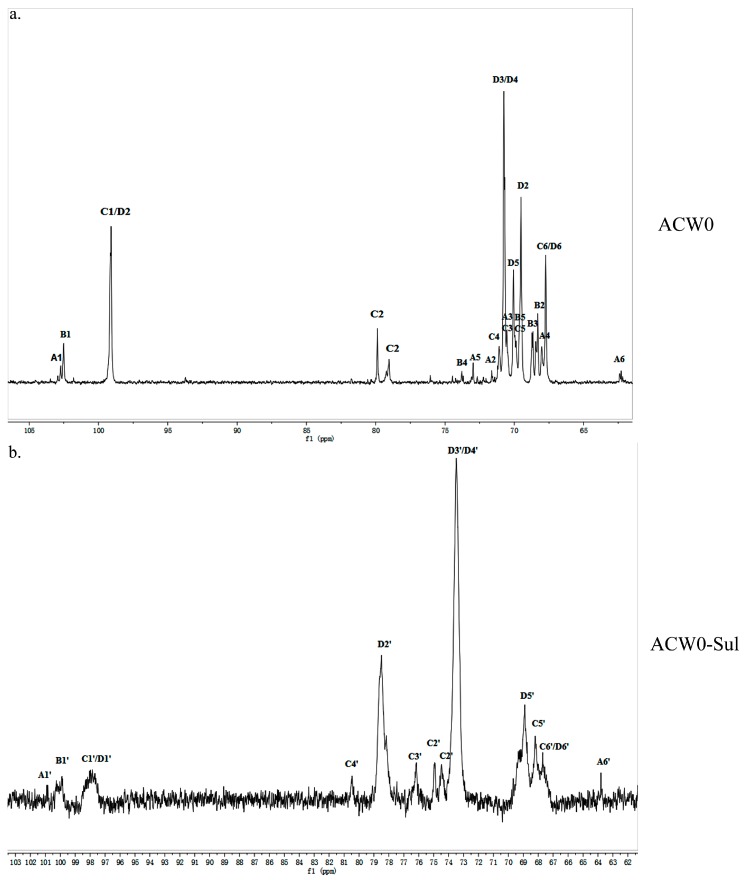
^13^C NMR spectrum of ACW0 (**a**) and ACW0-Sul (**b**). From the ^13^C NMR spectrum, we can see that δ 99.10 was from 1,2,6-linked galactose and 1,6-linked galactose, δ 102.45 was from terminal fucose, δ 102.73 was from terminal mannose. The chemical shifts at 60–80 ppm represented different ring carbon signal resonances. Among them, the C-6 atom of the unsubstituted hexose residue at δ 62.22 and C-6 signals at δ 67.76 for substituted pyranose residues were observed. Two signals at δ 79.08 and δ 79.80 indicated the substitution at C2–C5 of the sugar ring carbons. The methyl group (on C-6) of the fucose appeared at δ 16.99.

**Figure 4:**
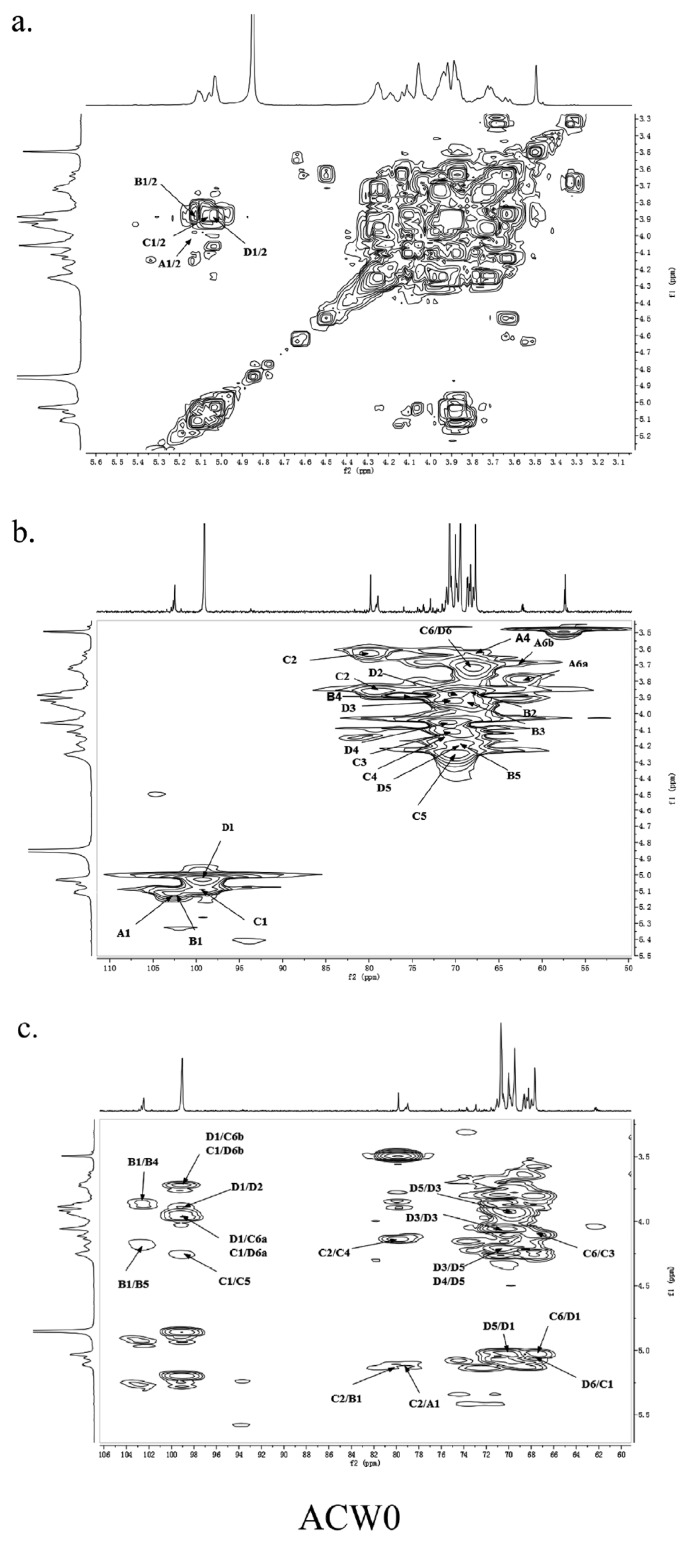
2D NMR spectra of ACW0. (**a**) ^1^H-^1^H COSY spectra of ACW0; (**b**) HSQC spectra of ACW0; (**c**) HMBC spectra of ACW0. In the picture, “A” represents terminally linked α-d-Man; “B” represents terminally linked α-l-Fuc; “C” represents 1,2,6-linked α-d-Gal; and “D” represents 1,6-linked α-d-Gal. The precise values include A1–6, B1–6, C1–6, and D1–6 are all in Table 2.

The structure of the polysaccharide, ACW0 was revealed with the help of NMR spectra, which are shown in Figure 2a, Figure 3a and Figure 4. As seen in ^1^H NMR, four anomeric proton signals appeared at δ 5.12, 5.11, 5.06, and 5.04, which suggested that all residues might have α configuration. In detail, the signals at δ 5.12 and δ 5.11 were attributed to terminal fucose and mannose, δ 5.06 from 1,2,6-linked galactose and δ 5.04 from 1,6-linked galactose, respectively [32]. Additionally, the ^13^C NMR spectrum contained three major anomeric carbon signals described as follows: δ 102.73 from terminal mannose, δ 102.45 from terminal fucose, δ 99.10 from 1,2,6-linked galactose and 1,6-linked galactose. The chemical shifts at 60–80 ppm represented different ring carbon signals resonances. Among them, C-6 atom of the unsubstituted hexose residue at δ 62.22 and C-6 signals at δ 67.76 for substituted pyranose residues were observed. Two signals at δ 79.08 and δ 79.80 indicated the substitution at C2–C5 of the sugar ring carbons. Besides, the methyl group (on C-6) of the fucose appeared at δ 16.99.

The other protons and carbon chemical shifts were further fully assigned based on previously reported data and 2D NMR spectra (COSY, HSQC, and HMBC) (Figure 4). The signals at 71.55, 70.47, 67.93, and 72.98 ppm corresponded to C2, C3, C4, and C5 of terminally linked mannose, respectively. Meanwhile, the weak signals at 68.35, 68.68, 73.78, and 69.87 ppm were then ascribed to C2, C3, C4, and C5 of terminal fucose, respectively. The downfield chemical shift at 79.80 ppm was from substituted C2 of 1,2,6-linked galactose. Besides, the weak resonance nearby at 79.08 ppm was also attributed to C2 of 1,2,6-linked galactose, which may due to the different chemical environment of 1,2,6-linked galactose caused by different substituted residues of non-reducing terminal mannose or fucose. Thus, other signals at 70.48, 71.10, and 69.88 ppm were assigned to C3, C4, and C5 of 1,2,6-linked galactose, respectively. Moreover, the remaining resonances at 69.50, 70.75, and 70.07 ppm were attributed to be C2, C3/C4, and C5 of 1,6-linked galactose, respectively. These data were in good accordance with previously reported results [15,18,33].

**Figure 5:**
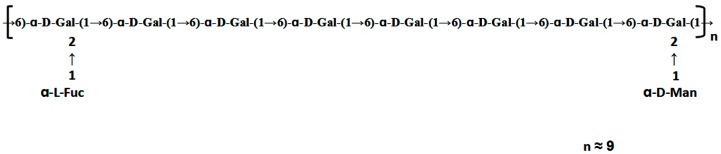
Proposed structure of ACW0.

The HSQC spectrum showed four cross peaks at 102.45/5.12, 102.73/5.11, 99.10/5.06, and 99.10/5.04 ppm representative of anomeric signals. Other correlation peaks were also assigned as indicated in Figure 4b. Long range correlation signals observed in the HMBC spectrum were used to presume the sequence of residues and further confirmed the results from HSQC. The inter-residue coupling between C2 at 79.80/79.08 ppm to H1 at 5.12/5.11 ppm identified the (1 → 2) linkage between 1,2,6-linked galactose and terminally linked mannose and fucose, respectively. Additionally, the cross peaks at 67.76/5.04 ppm and 99.10/3.94 (3.73) ppm indicated that C1 of 1,2,6-linked galactose was connected to C6 of adjacent 1,6-linked galactose, meanwhile those at 67.76/5.06 ppm and 99.10/3.94 (3.73) ppm indicated the linkage between C1 of 1,6-linked galactose and C6 of 1,2,6-linked galactose. All the above results revealed that the polysaccharide ACW0 has a backbone of 1,6-linked galactose with branches substituted at C-2 of 1,2,6-linked galactose. The complete assignments are shown in Table 2. Integration of the approximate molar ratio of methylation shows, two sugar residues, that is, terminal mannose and fucose were substituted at C-2 for nearly every six 1,6-linked galactose. Therefore, a possible proposed chemical structure for the repeating unit of polysaccharide ACW0 is illustrated below (Figure 5).

### 3.5. Preparation and Analysis of Sulfated Derivative of ACW0

The chlorosulfonic acid-pyridine method was used to modify the chemical structure of the polysaccharide ACW0 to obtain the sulfated derivative designated as ACW0-Sul [34]. Its specific optical rotation ${\left[\alpha \right]}_{D}^{25}$ is 62.3 (c = 0.1 g/100 mL). Monosaccharide composition analysis showed that it was composed of fucose (1.29%), mannose (7.49%), and galactose (91.23%), which were in agreement with native polysaccharide ACW0, indicating that the glycosyl residues of the polysaccharide were not changed after sulfation. The degree of sulfation was calculated to be 2.43 based on the content of sulfur with the barium chloride-gelatin method [35]. The IR spectra of ACW0 and ACW0-Sul are shown in Figure 1. Comparing with the IR spectra of these two polysaccharide, we found two strong absorption bands at 1259.85 cm^−1^ and 844.75 cm^−1^ of ACW0-Sul, which were attributed to S=O and C-O-S stretching vibration according to previous reported literature [36]. It is known that substituents such as sulfate groups cause regular shifts in the ^13^C NMR spectrum of signals of the nearest carbon atoms [37]. Hence, determination of the location of these substituents is possible. In the ^13^C NMR spectrum shown in Figure 3, the peak at 62.22 ppm representative of C-6 of the terminally linked mannose shifted towards lower field with peaks at 63.79 ppm, indicating that the C-6 of terminal mannose was substituted by a sulfated group. We also found that the signal at 70.75 ppm corresponding to C-3 or C-4 of 1,6-linked galactose shifted to 73.53 ppm as well as C-2 shifted from 69.50 ppm to 78.48 ppm, leading to adjacent C-5 at 70.07 ppm shifted towards higher field at 68.88 ppm. The results indicated that O-2, O-3 or/and O-4 of 1,6-linked galactose were sulfated to some degree. The signals at 70.48 ppm and 71.10 ppm, which were assigned to C-3 and C-4 of 1,2,6-linked galactose, also shifted towards lower field with neighboring C-5 at 69.88 ppm and C-2 at 79.80/79.08 ppm shifted towards higher field, suggesting sulfate substitutions at C-3 and C-4 of 1,2,6-linked galactose. The absorption at 844.75 cm^−1^ in the IR spectrum was assigned to the 4-sulfate of d-galactose [38], which was further confirmed by C-4 of 1,6-linked galactose and 1,2,6-linked galactose substituted by a sulfate group.

### 3.6. ACW0-Sul Inhibits HMEC-1 Tube Formation, Migration and Proliferation

**Figure 6:**
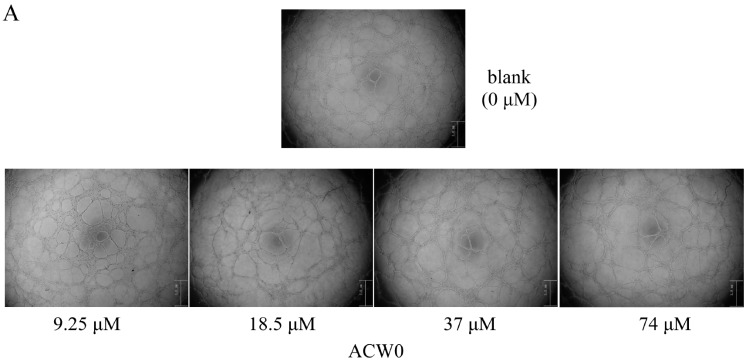

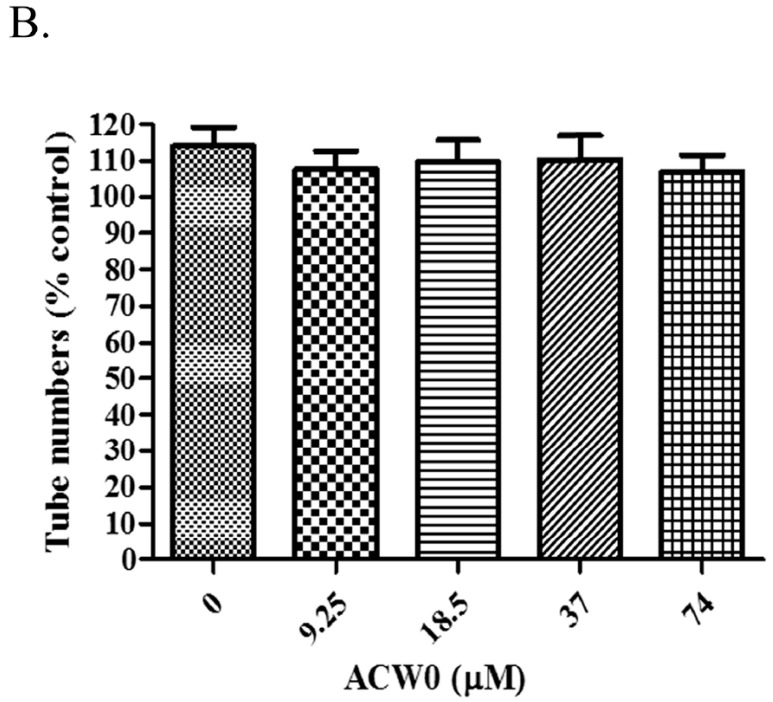
The effect of ACW0 on tube formation of HMEC-1 cells. (**A**) HMEC-1 cells treated with ACW0 at different concentrations (9.25, 18.5, 37, and 74 μM). The blank (0 μM) was control. The scale bar are 3.0 um. (**B**) Quantitative measurement of tube numbers. The values represent mean ± S.D.

**Figure 7:**
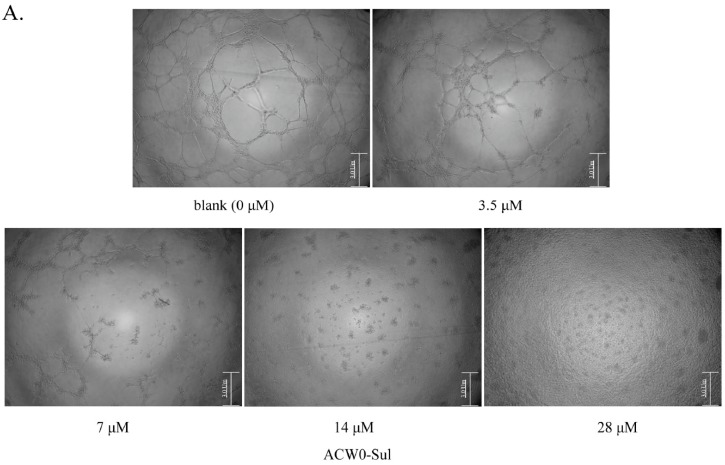

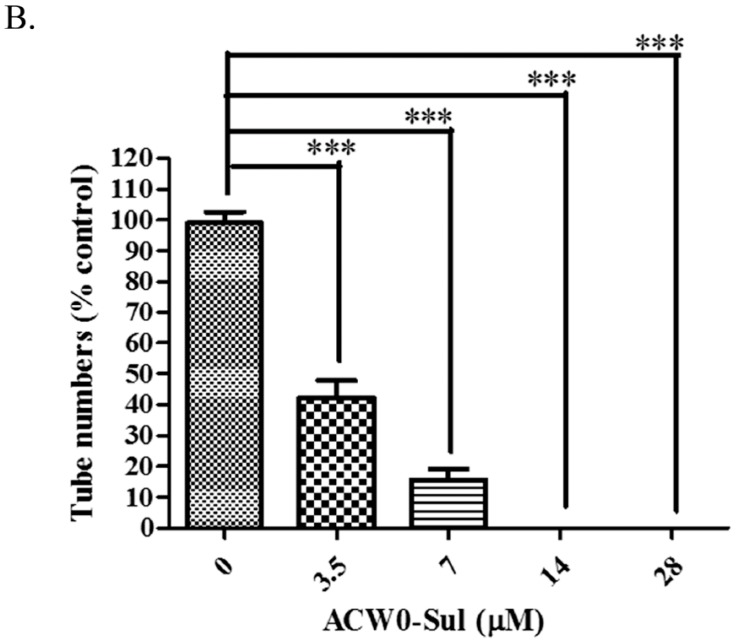
The effects of sulfated polysaccharide ACW0-Sul on tube formation of HMEC-1 cells. (**A**) HMEC-1 cells were treated with ACW0-Sul at different concentration (0, 3.5, 7, 14, and 28 μM). The scale bar are 3.0 um. (**B**) Quantitative measurement of (**A**). Each experiment was performed at least 3 times, and the values represent mean ± S.D. *** *p* < 0.001 as determined by unpaired *t*-test.

**Figure 8:**
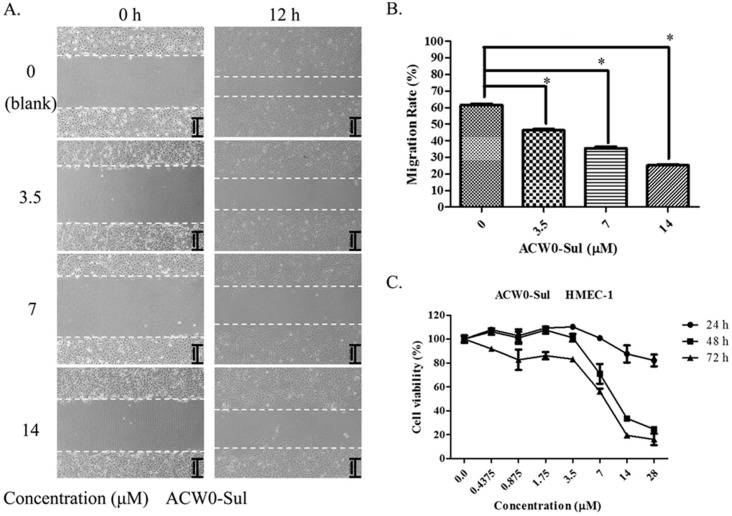
ACW0-Sul inhibited cell migration and cell proliferation of HMEC-1 cells. (**A**) The wound healing assay was performed with ACW0-Sul treatment at different concentrations (0, 3.5, 7, 14 μM) for 0 and 12 h. The wound areas at time 0 and 12 h are indicated by dotted lines. (**B**) Quantification of effect of ACW0-Sul on HMEC-1 cells migration in the wound healing assay. (**C**) HMEC-1 cells were treated with different concentrations of ACW0-Sul (0, 0.4375, 0.875, 1.75, 3.5, 7, 14, and 28 μM) for 24, 48, and 72 h, respectively, and followed by cell viability MTT assay. Each experiment was repeated at least 3 times, and the values represent mean ± S.D. * *p* < 0.05 as determined by unpaired *t*-test.

To investigate the inhibitory effects, tube formation assays in the presence of ACW0 (Figure 6) and ACW0-Sul (Figure 7) on Matrigel were performed using HMEC-1 cells. The results showed that only ACW0-Sul inhibited the tube formation dose dependently, and the effective concentration was about 3.5 μM (Figure 7). To determine whether ACW0-Sul can inhibit migration, HMEC-1 cells wound healing assay in vitro was also detected. To do this, close monolayer cells were damaged to impel cell migration into the wound area. After the HMEC-1 cells were treated with different concentrations of ACW0-Sul, substantial inhibition was observed (Figure 8A,B). The wound group 62.7% (±1.65%) in the absence of ACW0-Sul was closed after 12 h incubation. Nevertheless, only 46.3% (±1.25%), 35.18% (±2.09%) and 24.8% (±1.96%) of the wounds were enclosed after treatment with 3.5, 7 and 14 μM ACW0-Sul. These results suggested that ACW0-Sul significantly impeded the migration of HMEC-1 cells. 

To test if the inhibitory effects on capillary-like tube formation and migration were the consequence of inhibition of HMEC-1 cells proliferation or not, we monitored the viability of HMEC-1 cells. After treating the HMEC-1 cells with ACW0-Sul (0–28 μM) in vitro for 24, 48, and 72 h, As shown in Figure 8C, ACW0-Sul has no significant cytotoxicity effect on HMEC-1 cells at a concentration of 28 μM within 24 h, while the effective concentration on tube formation and migration we detected were only during 12 h. there was no significant inhibition on proliferation of HMEC-1 cells after 24 h treatment. However, the inhibition effect occurred when the treatment was longer. The cell viabilities were about 23% and 18% at 28 μM treatment for independently 48 h and 72 h. This data showed that the proliferation inhibition might also contribute to some extent to the anti-angiogenic function of ACW0-Sul. Taken together, these results suggested that ACW0-Sul could inhibit angiogenesis in vitro. The concrete details are accounted for in the former published literature [4].

### 3.7. ACW0-Sul Inhibits the Phosphorylation of Erk and FAK

**Figure 9:**
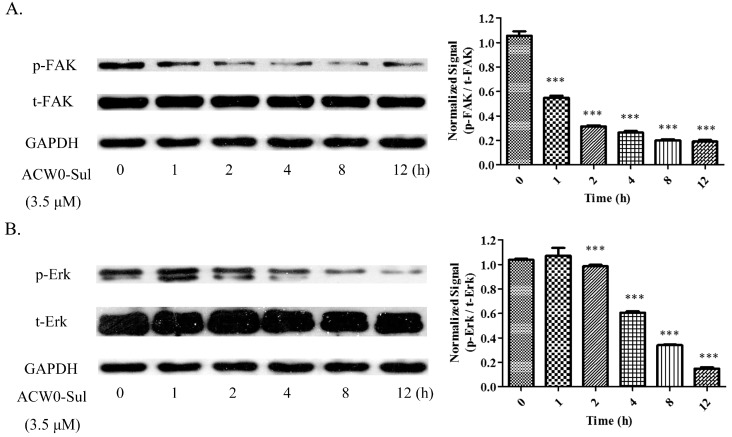
ACW0-Sul inhibited the phosphorylation of FAK and Erk. HMEC-1 cells incubated with 3.5 μM ACW0-Sul for different time (0, 1, 2, 4, 8 and 12 h). Then the cells were lysed and subjected to Western blotting measurement. (**A**) The expression of phospho-FAK and total FAK was measured by Western blotting. (**B**) The expression of phospho-Erk and total Erk was examined by Western blotting. GAPDH was used as loading control. The experiments were repeated at least 3 times, and the data were represented as mean ± S.D.; *n* = 3. *** *p* < 0.001.

To explore the underlying mechanism, phosphorylation of some key molecules was observed. Among them, the phosphorylation of Erk and FAK were attenuated significantly by ACW0-Sul after a different time treatment. However, activation of these signaling pathways is critical for angiogenesis [39]. A remarkable downregulation of Erk phosphorylation was measured with the treatment of ACW0-Sul (3.5 μM), whereas the expression of total-FAK was almost invariant (Figure 9A). Similarly, the phosphorylation of Erk (Figure 9B) was decreased after incubation with ACW0-Sul for 2 h. These results suggested that MAPK/Erk and FAK signaling pathways might play an important role in anti-angiogenesis through ACW0-Sul. These results are also consistent with previous reports [6,7].

## 4. Discussion

A heterogalactan, ACW0, isolated and purified from *Antrodia camphorate*, was shown to be a mannofucogalactan with a backbone chain of α-d-1,6-linked Gal, non-reducing terminal α-d-Man and α-l-Fuc substituted at O-2 for nearly every six α-d-1,6-linked Gal residues, composed of galactose (94.98%), traces of mannose (2.41%), and fucose (2.61%). The bioactivity test suggested that ACW0 has no inhibition effect on tube formation (Figure 6) while its sulfated derivative, ACW0-Sul, whose sulfate substitutions at C-3 and C-4 of 1,2,6-linked galactose, demonstrated a significant inhibition on tube formation and migration of HMEC-1 cells dose-dependently (Figure 7). This confirms that the sulfation group plays a key role in the angiogenesis inhibition effect. Further study showed that phosphorylation of Erk and FAK, which play an important part in the process of angiogenesis of proteins, were significantly inhibited by ACW0-Sul. These results successfully demonstrated the structure of a sulfated modification and the relationship between biological activities. The above results also suggested that ACW0-Sul might be a potential anti-angiogenic agent.

## Figures and Tables

**Table 1:** Linkage analysis of polysaccharide ACW0.

Methylated Sugars	Type of Linkages	Molar Ratios (%)	Mass Fragments (*m*/*z*)
2,3,4,6-Me4-Man	T-Manp	8.90	43,87,101,117,129,145,161,205
2,3,4,6-Me4-Fuc	T-Fucp	5.38	43,89,101,117,131,161,175
3,4-Me2-Gal	1,6-Galp	66.92	43,99,101,117,129,161,189,233
2,3,4-Me3-Gal	1,2,6-Galp	18.80	43,87,99,129,189,233

**Table 2:** ^1^H and ^13^C NMR assignments for ACW0.

Residues		1	2	3	4	5	6
A α-d-T-Manp	H	5.11	4.02	3.68	3.63	3.89	3.78/3.68
	C	102.73	71.55	70.47	67.93	72.98	62.22
B α-l-T-Fucp	H	5.12	3.86	3.92	3.89	4.20	1.29
	C	102.45	68.35	68.68	73.78	69.87	16.99
C α-d-1,2,6-Galp	H	5.06	3.86/3.63	4.11	4.14	4.25	3.94/3.73
	C	99.10	79.08/79.80	70.48	70.48	69.88	67.76
D α-d-1,6-Galp	H	5.04	3.89	3.92	3.92	4.19	3.94/3.73
	C	99.10	69.50	70.75	70.75	70.07	67.76
